# Lactoferrin as a Component of Pharmaceutical Preparations: An Experimental Focus

**DOI:** 10.3390/ph16020214

**Published:** 2023-01-31

**Authors:** Fabiola Guzmán-Mejía, Marycarmen Godínez-Victoria, Daniel Efrain Molotla-Torres, Maria Elisa Drago-Serrano

**Affiliations:** 1 Unidad Xochimilco, Departamento de Sistemas Biológicos, Universidad Autónoma Metropolitana, Ciudad de México CP 04960, Mexico; 2 Sección de Estudios de Posgrado e Investigación, Escuela Superior de Medicina, Instituto Politécnico Nacional, Ciudad de México CP 11340, Mexico

**Keywords:** lactoferrin, neurodegenerative diseases, intestinal bowel diseases, nanoparticles

## Abstract

Lactoferrin is an 80 kDa monomeric glycoprotein that exhibits multitask activities. Lactoferrin properties are of interest in the pharmaceutical field for the design of products with therapeutic potential, including nanoparticles and liposomes, among many others. In antimicrobial preparations, lactoferrin has been included either as a main bioactive component or as an enhancer of the activity and potency of first-line antibiotics. In some proposals based on nanoparticles, lactoferrin has been included in delivery systems to transport and protect drugs from enzymatic degradation in the intestine, favoring the bioavailability for the treatment of inflammatory bowel disease and colon cancer. Moreover, nanoparticles loaded with lactoferrin have been formulated as delivery systems to transport drugs for neurodegenerative diseases, which cannot cross the blood–brain barrier to enter the central nervous system. This manuscript is focused on pharmaceutical products either containing lactoferrin as the bioactive component or formulated with lactoferrin as the carrier considering its interaction with receptors expressed in tissues as targets of drugs delivered via parenteral or mucosal administration. We hope that this manuscript provides insights about the therapeutic possibilities of pharmaceutical Lf preparations with a sustainable approach that contributes to decreasing the resistance of antimicrobials and enhancing the bioavailability of first-line drugs for intestinal chronic inflammation and neurodegenerative diseases.

## 1. Introduction

Lactoferrin (Lf) is a monomeric glycoprotein with a molecular mass of 80 kDa belonging to the transferrin family. Lactoferrin is an iron chelator that binds reversibly two ferric (Fe^3+^) cations so that the iron free form is termed apolactoferrin (Apo-Lf), whereas fully saturated diferric Lf is known as hololactoferrin (Holo-Lf) [1,2]. Lactoferrin is a multifunctional glycoprotein that displays antimicrobial **[3]**, antiviral [4,5], immunomodulatory [6], and antioxidant properties [2]. Antimicrobial actions of Lf entail its microbiostatic effect (i) by preventing the uptake of iron as an essential factor for the growth of some microbial species and (ii) by interacting with the surface components of bacteria, protozoans, and yeast, resulting in an increased permeability, disruption, and structural damage of the microbial surface [3]. Lactoferrin displays antiviral properties to influenza viruses, herpes simplex viruses, the hepatitis C virus, coronaviruses, and retroviruses [3,5]. Lf has antiviral action by inducing innate antiviral immunity to the norovirus causing gastroenteritis as documented in B cell culture assays [4]. The underlying mechanisms entail the ability of Lf to bind the virus envelope protein or the virus receptor and to block the virus entry to host cells or indirectly to prevent virus-induced apoptosis [3]. Experimental studies support the antiviral action of Lf, although the antiviral outcome of Lf is not so clear as documented in human trials [5]. 

Lactoferrin acts as a link between innate and adaptive immunity. Lf enhances the generation of the antibody response and modulates the interleukin generation and the activation of cells responsible for adaptive immunity, i.e., antigen-presenting cells. Lf modulation proceeds across the cell surface through receptor signaling and/or via internalization resulting in modulation of transcriptional nuclear factors [6]. Lactoferrin participates in the regulation of oxidative stress that either plays a critical role in protection against numerous microbial infections or has a detrimental impact on chronic degenerative processes [3]. Lactoferrin’s ability to bind reversibly free Fe^3+^ cations helps to balance iron levels in the body [6]. An excess of free Fe^3+^ cations can be toxic because they can donate electrons to diatomic oxygen (O_2_) necessary in the formation of reactive oxygen species (ROS). It is known that the rate and extent of ROS generation and removal are dependent on the enzymatic efficiency of superoxide dismutase (SOD), glutathione peroxidase (GPx), and catalase (CAT). Ferric cations drive the generation of ROS including superoxide radical (·O_2_−), hydroxyl radical (·OH^−^), and hydrogen peroxide (H_2_O_2_) via Fenton reaction; the latter encompasses the participation of the enzymes SOD, GPx, and CAT. Thus, by binding Fe^3+^ cations, Lf is able to prevent ROS production and avoid the deleterious effects of oxidative stress [2].

Lactoferrin can carry out some modulatory actions due to its interaction with multiple eukaryotic receptors expressed on a wide array of target cells. Receptors that bind to Lf include intelectin-1 (omentin-1), CD14, chemokine receptor 4 (CXCR4), and low-density lipoprotein receptor-related protein (LRP), among many others. Intelectin-1 is expressed in the small intestine and enables Lf uptake, that is, the binding and endocytosis of Lf; CD14 and CXCR4 are found in phagocytic cells; and LRP is distributed in the nervous system [7].

Basic knowledge of the multifunctional profile of Lf is of high interest in the pharmaceutical field for the design of products for potential applications in clinical practice. At present the pharmaceutical formulations containing Lf include microparticles, nanoparticles, emulsions, liposomes, and some others. These products have only been tested in experimental studies; therefore, their assessment in human trials remains to be accomplished.

This manuscript is focused on pharmaceutical products either containing Lf as a bioactive therapeutic component, considering its antimicrobial, antiviral, antioxidant, and immunomodulatory properties, or designed with Lf as a carrier considering its interaction with receptors in tissues and organs as targets of drugs delivered via parenteral or mucosal administration. We hope that this manuscript provides insights about the therapeutic possibilities of pharmaceutical Lf preparations a sustainable approach that contributes to decreasing the resistance of antimicrobials and reducing the tolerance of first-line drugs for chronic diseases. This contribution displays an overview of pharmacokinetics and pharmacodynamics properties of Lf formulations with potential applications for the treatment of microbial infections (bacterial, protozoan, and virus), intestinal inflammatory dysfunctions, and neurodegenerative diseases. 

## 2. Pharmacologic of Lactoferrin Formulations: General Aspects

Lactoferrin is used for many pharmaceutical and food applications, and their functions depend on structure and conformation. In this section we will address some pharmacokinetic (absorption and biodistribution) and pharmacodynamic aspects that have led to the use of Lf in the development of different carrier systems. These carrier systems are especially important for the use of formulations that require (i) improving the stability of Lf to be administered orally, alone or in combination with other drugs sensitive to degradation in the gastrointestinal tract (GIT) and (ii) driving drugs into the CNS that cannot cross the blood–brain barrier (BBB) by taking advantage of Lf receptor-mediated cellular capture processes.

The oral route of administration is the most convenient, safe, and economical route. To achieve therapeutic efficiency, an orally administered drug must be absorbed in the GIT to be bioavailable in plasma and be distributed through the bloodstream to target organs. Orally administered Lf and Lf-peptide derivatives experience numerous challenges such as inactivation by pH values and enzymatic degradation by GIT peptidases [8]. Therefore, carrier systems are used for improving the stability and bioavailability of Lf or different drugs that are sensitive to gastric degradation when they are administrated orally. The most important formulation approaches for bioactive proteins are lipid-based nanocarriers such as oil-in-water nanoemulsions, self-emulsifying drug delivery systems (SEDDS), solid lipid nanoparticles (SLN), nanostructured lipid carriers (NLC), liposomes, and micelles [9]. Proteins in combination with lipid components of carrier systems form highly lipophilic structures in which peptidases and hydrolases cannot act, thus protecting the bioactive protein [10,11]. For the intestinal absorption process, lipid-based nanocarriers are internalized by endocytosis and transcytosis via a clathrin-dependent endocytic pathway [9].

Many experimental approaches have been made to evaluate how different carrier systems, such as microparticles or nanoparticles and liposomes, can improve the stability of Lf. These oral delivery systems focused on the stability of Lf recently have been extensively reviewed [12,13,14]. Using an in vitro simulated digestive model, it has been shown that bLf is sensitive to gastric digestion [15], but when it is formulated in oral carriers, the systems’ proteolysis is decreased [16,17,18,19,20]. This oral delivery system showed high stability in gastric conditions and effectively protected Lf from digestion and improved the intestinal absorption. Lactoferrin absorption is mediated by the existence of receptors (LfR) expressed in different tissues [7]. In the GTI, intelectin-1 receptor is responsible for transporting Lf within enterocytes as it has been reported on Caco-2 epithelial cell cultures. Immunochemical assays showed the co-localization of Lf and intelectin receptor within endosomes marked with endosome antigen 1 (EEA1) [21]. Studies have shown that Lf in carrier systems improves the cell uptake vs. Lf native or non-formulated on Caco-2 cells cultures [22,23]. Once captured by epithelial cells, Lf can then be released intact or fragmented into the culture medium [24]. These findings suggested that at the intestinal level, lactoferrin is endocytosed to be released at the lamina propria. 

Lf present in the lamina propria can be distributed to the blood circulation via the lymphatic system [25,26]. A study evaluated the amount of bLf or bLf liposomes administered intraduodenally to Wistar rats; it was found that both formulations had the same absorption profiles evaluated as plasma concentrations of Lf [26]. This assay indicates that Lf formulations do not alter the absorption process in epithelial cells, but the Lf administrated intraduodenally does not allow the observation of the effect of gastric degradation of Lf.

In vivo assays of biodistribution in mice fed Lf nanoparticles (Lf-NPs) in their diet showed that the amounts of Lf in different tissues such as the stomach, small intestine, large intestine, heart, liver, lung, and brain was found to be greater than in mice fed a diet without a delivery system [22,27,28]. Having in mind the efficient biodistribution of Lf NPs within the brain, experimental studies addressed the use of Lf as a drug carrier targeted to the CNS via LfR. Lactoferrin receptor low-density lipoprotein receptor-related protein 1 (LRP1) has been reported in the brain including the blood–brain barrier (BBB) [29,30]. Binding of Lf to the LRP induces the transport of Lf through brain endothelial cells within the central nervous system (CNS) [31]. Some Lf nanoformulations have been designed as transporters of drugs that do not cross the BBB to the CNS for Alzheimer’s and Parkinson’s treatment. Experimental biodistribution assays in mice treated intravenously or intranasally with fluorescent Lf–drug nanoparticles showed their accumulation in the brain [32,33]. The data suggested the efficient BBB crossing of Lf NPs and underlined their potential use for the transport of drugs in the CNS. 

## 3. Antimicrobial Products

A very serious concern has been raised by the misuse and overuse of antimicrobials to combat pathogenic microorganisms resulting, in part, from auto-medication by users that dismiss the prescription and ignore that the antibiotic treatment is not recommended in some infectious diseases, for example, enterohemorrhagic diarrhea caused by Enterotoxigenic *Escherichia coli* 0157:H7 (EHEC 0157:H7) [34]. Failure in the therapeutic approach demands not just the administration of potent cocktails prepared by combinations of several antimicrobials but also the increasing dosage needed for reaching an effective eradication of pathogens. Regrettably, this approach favors (i) the ensuing selection of “superbugs,” i.e., multidrug-resistant microbial pathogens; (ii) the killing of beneficial microbiota, (iii) collateral threats (anaphylaxis); and (iv) drug toxicity (liver, kidneys, and ear, among others [35,36]. Moreover, antimicrobial application is a usual prophylactic practice in some other fields including the food industry, leading to environmental issues [37]. The misuse and overuse of antimicrobials have propelled sustainable approaches based on the design of antimicrobials containing Lf to reduce the dosage and to enhance the antimicrobial effectivity of standard drugs and in some cases to be used as alternatives to replace the use of conventional antimicrobial applications and reducing their side effects. Moreover, formulations containing Lf as a natural antimicrobial agent or as a carrier may be a promising and sustainable strategy. Although experimental studies suggested robust insights, future studies in human trials are needed to confirm the effectivity and the toxicity of the lactoferrin products. Information concerning antimicrobial mechanisms, bioavailability, and/or toxicity is not provided to weight the advantages and disadvantages of these products. The next section describes some formulations containing Lf analyzed in preclinical studies and summarized in Table 1. 

### 3.1. Antibacterial Products

*Salmonella enterica* serovar Typhimurium (*S*. *typhimurium*) is a Gram-negative bacteria causing self-limiting gastroenteritis in humans and enteric fever in mice that resembles the enteric fever infection caused by *Salmonella typhi* in humans [49]. The morbidity and mortality of salmonella infections result from the multidrug-resistant ability of salmonella strains to evade conventional antibiotics such as ciprofloxacin [50]. Thus, the antimicrobial outcome of Lf loaded in alginate gel-encapsulated ceramic nanocapsules (NCs) was tested in the murine model of typhoid fever [38]. The experimental protocol was conducted in BALB/c mice infected by gavage with *Salmonella typhimurium* and thereafter fed with 1.2% *w*/*w* iron-saturated Lf-NCs formulated in a pellet diet. The findings indicated that NCs loaded with Lf accelerated the bacterial clearance in the intestine and decreased the bacterial translocation in the liver and spleen. Regarding the ciprofloxacin as a positive antimicrobial control, treatment with Lf-NCs accelerated the elimination avoiding chronic infection. The mechanism accounting for the antisalmonella activity was related to an efficient uptake via a receptor and improving the endocytosis, efficient biodistribution, and the enhancement of immunity involved in the resolution of the salmonella infection [38]. 

*Helicobacter pylori* is a Gram-negative bacteria that inhabits the gastric mucosa and is a leading cause of chronic gastritis that eventually drives gastric ulcers, duodenal ulcers, and gastric adenocarcinoma [51]. Antibiotic treatment faces an increasing antibiotic resistance to first-line antibiotics including metronidazole, amoxicillin, and/or clarithromycin [51]. An alternative product based on Lf loaded in biomimetic hydroxyapatite nanocrystals combined with the cell free supernatant from *Lactobacillus paracasei*, a probiotic with beneficial effects on gut health, has been proposed as a therapeutic for *Helicobacter pylori* infection [39]. The experimental setting was conducted in BALB/c mice orally infected with *Helicobacter pylori*, and thereafter, mice received three doses of Lf loaded in hydroxyapatite NPs (300 μg/mouse) and the probiotic cell free supernatant (50 μL/mouse). The findings indicated that with regard to the antibiotic mixture containing amoxicillin and clarithromycin (each one being 300 μg/mouse), the gastric bacterial load resulting from the treatment with Lf-hydroxyapatite NPs plus probiotic cell free supernatant was lower after the first week or similar after three weeks [39]. Like the antibiotic mixture treatment, the antibacterial activity of the Lf hydroxyapatite NPs plus probiotic cell free supernatant was accompanied by the amelioration of proinflammatory cytokine generation and the enforcement of anti-inflammatory cytokines, antibody IgA responses, and reduced histopathological gastric damage [39].This alternative approach may provide some advantages over antibiotic treatment associated with *Helicobacter pylori* antibiotic resistance and collateral tissue damage. Findings about the potential toxicity of Lf hydroxyapatite NPs were not provided in this contribution. 

*Porphyromonas gingivalis* is a Gram-negative oral anaerobe belonging to oral microbiota, and under oral mucosa perturbations, it becomes a pathobiont that proliferates, causing severe and persistent periodontal lesions; *Porphyromonas gingivalis* has developed antibiotic resistance due in part to its ability to form biofilms [52]. In this regard, pharmaceutical preparations containing Lf loaded in liposomes have been proposed for gingivitis treatment as documented in human volunteers with periodontal infection [40]. Human subjects with 4 mm probing depth were selected, and they were asked to take four tablets of liposome containing 180 mg bLf as a supplement per day for 4 weeks; periodontal examination included the assessment of probing depth, bleeding on probing, and sampling of gingival crevicular fluid. Lactoferrin loaded in liposomes formulated in tablets significantly decreased the probing depth with no effect on bleeding on probing after 4 weeks of treatment. Moreover, the Lf liposome tablets decreased the monocyte chemoattract protein-1 (MCP-1), and the lipopolysaccharide induced cytokine production in peripheral blood mononuclear cell cultures. Future large-scale trials in humans may confirm the therapeutic effectivity to combat periodontal infections associated with *Porphyromonas gingivalis* [40].

### 3.2. Antiparasite Products

*Toxoplasma gondii* is an obligate intracellular protozoan parasite causing zoonosis of veterinary and medicine relevance. *Toxoplasma gondii* is harbored by cats as a definitive host and by birds, rodents, and humans as intermediate hosts. *Toxoplasma gondii* exhibits a very complex life cycle including a fast-replicating stage as a tachyzoite and a low-growing stage as bradyzoites that ultimately generate cysts. *Toxoplasma gondii* can be transmitted from mother to fetus to cause congenital malformations. *Toxoplasma gondii* causes persistent infection in the brains of immunodeficient individuals and unborn fetuses [53]. Given its intracellular habitat, the eradication of *Toxoplasma gondii* demands an aggressive treatment that, to be effective, requires a cocktail of strong antiparasitic and toxic drugs such as pyrimethamine combined with sulfadiazine or clindamycin or alternatively trimethoprim and sulfamethoxazole [54]. Antiparasitic action bLf loaded as a therapeutic in AEC-CCo-CP-NCs delivered in pellet food was tested in BALB/c mice infected with *Toxoplasma gondii* tachyzoites [27]. Data indicated that with regard to mice fed with pellets containing purified bLf, mice fed a diet containing bLf-AEC-CCo-CP-NCs exhibited greater generation of reactive oxygen species, nitric oxide, and Th1 cytokine profiles along with lower parasite load and higher parasite clearance in the liver and spleen. Assays of tissue distribution indicated that the bLf concentration loaded in NPs was greater in tissues with regard to purified bLf. The findings suggested that NPs increased the bioavailability and stability of bLf, resulting in greater antiparasitic activity, although toxic effects of these Lf-NPs remain for further studies. Antiparasitic activity of Lf -AEC-CCo-CP-NPs may provide advantages over conventional drug therapy. 

*Leishmania donovani* is a protozoan parasite that causes persistent visceral leishmaniasis, known also as Kala azar, transmitted via the bite of phlebotome sand flies as the preferred vector [55]. The *Leishmania donovani* life cycle entails a promomastigote found in the vector and amastigote found in the host. Amphotericin B, the first choice for antiprotozoan treatment, is highly toxic and eventually loses efficiency because of the development of protozoan resistance [55]. 

To potentiate the antileishmaniasis action, amphotericin B was encapsulated inside Lf-appended poly D,L-lactide-coglycolide nanoreservoir (LcfPGNP-AmB) and tested in hamsters infected with *Leishmania donovani* amastigotes [41]. With regard to AmB alone, LcfFPGNP-AmB showed no toxicity and had potent antileishmania action by decreasing the parasite load in the spleen. LcfPGNP-AmB was mostly distributed in the spleen and increased a Th1 response associated with the release of the proinflammatory cytokines interleukin (IL)-12, interferon-γ (IFN-γ) and tumor necrosis factor-α (TNF-α) for the protozoan clearance; efficient uptake and endocytosis of LcfPGNP-AmB was seen in macrophages intracellularly infected with promastigotes. The results indicate that LcfPGNP-AmB action provides potent antileishmanial effect and lower toxicity than amphotericin B, which is highly toxic. The antiparasite action was attributed to the efficient uptake of an Lf-appended nanoreservoir through receptor mediated endocytosis via C-type lectin and the intracellular AmB release.

A product formulated with betulinic acid, a pentacyclic triterpenoid from Betula alba bark entrapped in PLGA NPs coated with bLf (Lf-BANPs), has been tested in in vitro cultures of macrophages isolated from BALB/c mice infected with *Leishmania donovani* [42]. In this assay, the Lf-BANP treatment decreased amastigote count in macrophages to a higher extent than the betulinic acid or BANPs. Moreover, Lf-BANPs increased the nitric oxide associated with leishmania eradication and ameliorated IL-10 production that favored the leishmania infection 

*Plasmodium berghei* is a protozoan parasite causing malaria, a vector-borne transmitted infection via the bite of *Anopheles* mosquito that is highly prevalent in tropical and subtropical countries [56]. The *Plasmodium berghei* life cycle entails a host stage where the schizont proliferation within erythrocytes and the release and spreading via the bloodstream of infective merozoite to the liver and spleen take place [57]. The effective treatment of malaria entails highly toxic chloroquine and sulfadoxine that can be limited and even ineffective due to the antimalarial drug resistance that arises by mutations during sexual parasite stages in the mosquito [56]. Antimalarial outcomes of alginate-enclosed, chitosan-conjugated, calcium phosphate buffalo Lf-NCs (AEC-CCo-CP-buLf-NCs) containing iron-saturated buLf 1.2% *w*/*w* were tested in a murine model of malaria [43]. An experimental study was conducted in BALB/c mice infected via intraperitoneal injection with parasitized erythrocytes from Swiss albino mice, and for three days post-infection, the mice were fed AEC-CCo-CP-buLf-NCs in their diet [43]. AEC-CCo-CP-buLf-NCs were efficiently distributed in the blood, intestine, liver, and spleen; moreover, AEC-CCo-CP-buLf-NCs exhibited effective antimalarial action evidenced by a significant decrease in parasite load in erythrocytes and reduced damage in the liver and spleen as seen with chloroquine, the antimalarial drug of reference, which was included as a positive control on day 12 post-infection. Furthermore, AEC-CCo-CP-buLf-NC treatment favored a survival rate of 100% as found with chloroquine until day 35 post-infection. The antimalarial outcome of AEC-CCo-CP-buLf-NCs was also evidenced by the elicitation of a robust response of mediators for killing of phagocytosed parasites such as free iron radical production associated with a decreased Fab1 gene parasite qPCR replication and increased production of innate and adaptive cytokines involved in the parasite clearance [43]. 

### 3.3. Antiviral Products

Herpes simplex virus type 2 (HSV-2) is a leading causative agent of sexually transmitted genital ulcers and is regarded as a relevant health concern in developed and developing countries [58]. HSV-2 carries out latent establishment at spinal sacra ganglia and causes painful ulcers that can be recurrent, although they are asymptomatic in some subjects; for treatment, specific competitive viral DNA polymerase inhibitors (acyclovir, valacyclovir, and famciclovir) are prescribed, although the antiviral drugs neither eradicate the latent virus nor prevent the severity or recurrence after the drug treatment [58]. In this regard, the anti-HSV-2 activity of human recombinant iron-saturated Lf loaded in silver and gold NPs (Lf-Au-Ag-NPs) was tested in a murine model of HSV-2 infection. The experiments were conducted in female C57BL/6 mice infected intravaginally with HSV-2, and after the infection, vaginal washings with 10 μg/mL Lf-Au-Ag-NPs in saline solution were accomplished. The antiviral effectivity of Lf-Au-Ag-NPs was evidenced by a significant reduction of viral titers in vaginal fluids and tissue samples from the vagina and spinal cord collected on day 7 post-infection. Treatment with Lf-Au-Ag-NPs triggered the humoral (IL-1β, TNF-α, and IFN-γ) and cellular (natural killer cells, CD8+T cells, and dendritic cells) responses essential for the resolution of viral infection. Accordingly, the action of Lf-Au-Ag-NPs on HSV-2 vaginal infection may be an alternative or an adjunct therapy of antiviral drugs for HSV-2 genital infections [44]. 

Human immunodeficiency virus (HIV) is the causative agent of the acquired immunodeficiency syndrome (AIDS), a chronic illness transmitted sexually, among other routes [59]. HIV is a retrovirus characterized by encoding both reverse transcriptase for the transcription of RNA genomes to linear double-stranded viral DNA and the viral protein integrase critical for the integration of DNA associated with the viral/host nucleoproteins to the host cell genome [59]. The treatment for HIV/AIDS includes a combination of nucleotide reverse transcriptase inhibitors (for example, zidovudine (AZT) and tenofovir), protease inhibitors (for example, efavirenz), and integrase inhibitors (cabotegravir, among others); the antiretroviral drug treatment is ineffective due to the development of HIV resistance of these drugs, which are highly toxic. Therefore, the design of drugs that are highly effective for HIV infection and have low toxic effects has propelled several studies. 

**Figure 1:**
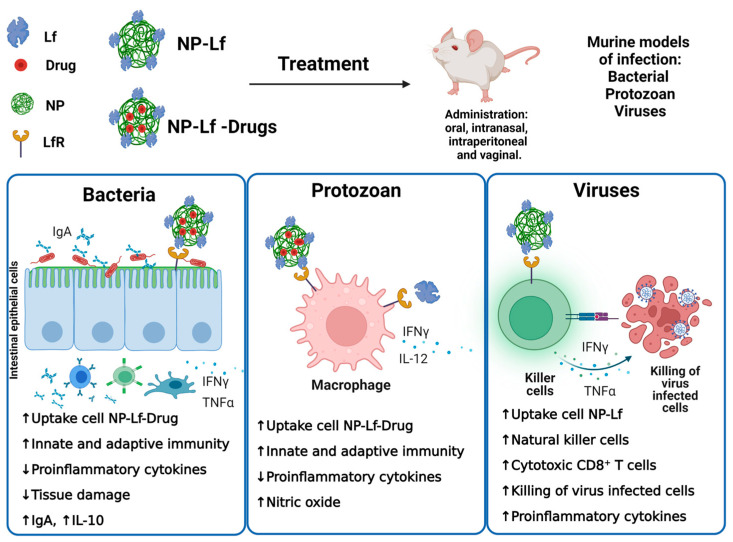
Lactoferrin products and antimicrobial effects in pathogenic bacteria, protozoans, and viruses. Created in Biorender.com. accessed on 24 January 2023.

The formulation of zidovudine (AZT) in Lf-NPs administered by an oral route in rats showed efficient bioavailability, stability at the low pH of the stomach, twofold lower toxicity, and pharmacokinetic improvement compared with AZT alone [45]. Some preparations designed with a multipurpose technology have been formulated with Lf-NPs as vehicles loaded with efavirenz (E) and curcumin (C), a pleiotropic turmeric herbal compound with potent antioxidant and anti-HIV activities [46]. The analysis of the intravaginal administration in rats evidenced that with regard to each single component, EC-Lf-NPs showed higher delivery in vaginal mucosa and lower absorption in the vagina and plasma; the area under the curve (AUC) and other pharmacokinetic parameters suggested longer vaginal exposure. Moreover, EC-Lf-NPs reduced the response of the pro-inflammatory cytokines IL-6 and TNF-α in vaginal tissue and plasma and further displayed a lower toxicity in vaginal tissue and did not affect the viability of beneficial strains such as the *Lactobacillus* member of the vaginal microbiota [46]. Additional assessments of EC-Lf-NPs and other NPs loaded with antiretroviral drugs and curcumin supported the vagina as the preferred route of delivery of these antiviral products with no effects on the reproductive performance, fertility, and postnatal development as documented in rats [47]. Additional formulations of Lf-NPs loaded with tenofovir, a reverse transcriptase inhibitor, and curcumin (TCNPs) showed efficient adhesion in the vaginal mucosa and minimal leakiness into the blood circulation; TCNPs displayed potent HIV-microbicide action along with low toxicity without inducing vaginal mucosa inflammation [48]. An overview of the antimicrobial mechanisms is depicted in Figure 1.

## 4. Intestinal Dysfunctions

Inflammatory bowel disease (IBD) comprises Crohn’s disease and ulcerative colitis (UC), two chronic and incurable inflammatory pathologies that affect millions of people worldwide [60]. Crohn’s disease predominantly affects the deep layers of the intestinal wall of the small intestine, although it can also affect the large intestine. Ulcerative colitis is a complex intestinal disease characterized by inflammation, destruction of the intestinal barrier, and an exaggerated immune response that conditions the formation of ulcers and is limited to the colon and rectum; long-term UC is a risk factor for colorectal cancer [60]. For the therapeutic IBD treatment, several anti-inflammatory agents are prescribed such as amino salicylates (5-aminosalicylic acid) and non-systemic steroids (budesonide); however, these agents are not effective with long-term use [61]. Therefore, novel therapeutic approaches with anti-inflammatory action are under analysis [60], and some of them have been focused on Lf as a natural anti-inflammatory protein [62,63]. In vitro culture assays evidenced that native bovine Lf (10.2 ± 0.2% iron saturation), Apo-Lf (1.2 ± 0.2% iron saturation), Holo-Lf (71.8 ± 6.5% iron saturation), and MnLf (47.1 ± 2.0% manganese saturation) induced higher levels of apoptosis or necrosis in Caco-2 cells, and all Lf forms inhibited the secretion of pro-inflammatory cytokines in the LPS-primed murine J774A.1 cell line. Native Lf did not stimulate the secretion of lipopolysaccharide (LPS)-induced cytokines (interleukin (IL)-1β, IL-6, tumor necrosis factorα (TNFα), and IL-10) in Caco-2 epithelial cells and the murine macrophage J774A.1 cell line. High concentrations of all forms of Lf did not alter the tight junctions in the Caco-2 monolayer. The data suggested that Apo-Lf has a potent inhibitory effect on the LPS-dependent activation of the macrophages, and it was able to sequester iron and inhibit the growth of pathogenic bacteria [62]. The data suggested that these effects may result from its ability to enhance the LfRs’ expression on enterocytes and their saturation to neutralize the LPS-induced inflammation. All forms of Lf tested in this study may have a beneficial impact on gut homeostasis due to prebiotic properties toward *Lactobacillus* strains. 

Dextran sodium sulfate (DSS)-treated C57/BL6 mice as a murine model of colitis and TNF^∆ARE/+^ mice as a murine model of Crohn´s disease-like ileitis addressed the anti-inflammatory properties of recombinant human Lf (VEN-120) administered at 500 mg/kg/14 days by gavage [63]. In vitro culture assays of lamina propria (LP) lymphocytes from TNF^∆ARE/+^ mice evidenced that VEN-120 decreased TNFα, interferon γ (IFNγ), and IL-6 TCD4+ cells and increased IL-10 TCD4+ cells. In addition, in cell cultures of LP, mesenteric lymph nodules (MLN), and spleen from DSS-treated mice and TNF^∆ARE/+^ mice, VEN-120 changed the Th17 cell profile to Treg FoxP3+/IL-10+ TCD+ cells. Furthermore, histopathologic examination of both DDS-treated mice and TNF^∆ARE/+^ mice evidenced that the administration of VEN-120 decreased the shortening of the colon and the inflammatory score and improved the intestinal histological architecture, decreasing the tissue injury, inflammatory infiltrate, loss of crypts, and epithelial integrity of small intestine and colon and increasing the goblet cells. The led to the conclusion that VEN-120 promotes a regulatory T-cell phenotype and drives the resolution of inflammation in colitis and ileitis murine models of IBD [63].

The attenuation of inflammation and the promotion of intestinal mucosa repair are the desired outcomes of novel drugs for treatment of UC [22,64,65]. As stated above, for IBD treatment, the frequently prescribed and most effective drugs include amino salicylates (5-aminosalicylic acid) and non-systemic steroids (budesonide) [61]; however, adverse side effects to long-term use and exacerbations of the disease limit their use. 

Several alternative products containing Lf have been designed for potential therapeutic approaches to IBD and cancer colon, as shown in Table 2. 

### 4.1. Inflammatory Bowel Diseases: Ulcerative Colitis and Crohn’s Disease

Preparations containing Lf have been formulated with anti-inflammatory compounds from plants such as emodin (EMO1,3,8-trihydroxy-6-methyl-9,10-anthraquinone); EMO is a bioactive component found in polygonaceous plants [22]. These herbal derivatives administered by the oral route have as their main limitations poor water solubility, low bioavailability, and poor targeting ability toward UC lesions [22]. Therefore, a nano-in-micro-oral delivery system based on emodin (EMO) encapsulated with Lf to form nanoparticles (EMO-NPs) and then loaded into yeast cell wall microparticles (YPs) was prepared to provide a final formula with two outer–inner targeting layers. This formulation of EMO-NYPs tested by scanning microscopy and fluorimetric cytometry indicated an efficient uptake via dectin in RAW-264.7 macrophages and through the Lf receptor in Caco-2 epithelial cells. An in vivo model in DSS-induced UC in BALB/c mice indicated that EMO-NPs enhanced the anti-inflammatory effect by inhibiting the toll-like receptor 4 (TLR4)-linked nuclear factor κB (NF-κB) signaling pathway and p65 expression as well mucosal repair by inhibiting the myosin light chain kinase/phospho-myosin light chain 2 (MLCK)/pMLC2 signaling pathway. In DSS-induced colitis in mice, EMO-NYPs increased the structural integrity of the colon, the number of goblet cells, and zonula occludends-1 (ZO-1), occludin, and claudin-1 expression to promote intestinal mucosa repair [22]. Furthermore, EMO-NPs decreased weight loss, shortening of the colon, the DAI score, intestinal tissue injury, and inflammatory infiltrate. The data suggested the EMO-NYPs displayed an efficient uptake by oral delivery with potential treatment for ulcerative colitis. 

In other contributions, calcium pectinate (CP) and hyaluronic acid (HA) modified Lf nanoparticles (NPs) were used as delivery systems for loading Rhein (RH), an herbal compound with anti-inflammatory properties, and tested in DSS-treated mice as a model of ulcerative colitis. This oral CP-HA-RH-NP delivery approach was designed to protect the Rhein against degradation by gastrointestinal enzymes and to be targeted to the colon; after arriving at the colon, the CP contained in the CP-HA-RH-NPs is degraded by microbiota enabling the HA-RH-NPs to release at the lesion site. In vitro culture assays in RAW 264.7 macrophage and Caco-2 cells evidenced that CP-HA-RH-NPs favored the HA-NP uptake. In vivo experiments in DSS-induced colitis in mice treated with CP-HA-RH-NPs showed that RH decreased the proinflammatory cytokine (IL-1β, TNFα, and IL-6) levels and inducible nitric oxide synthase (iNOS) release through the TLR4/myeloid differentiation 88 adaptor protein (MyD88) TLR4/MyD88/NF-κB pathway and increased the ZO-1 and claudin-1 expression in the colon. The data suggested that Lf is an efficient ligand in the delivery system to promote the effect of RHs in the colonic repair for colitis treatment [23].

In additional experimental studies, Lf loaded in nanoparticles was used as a delivery system in order to address the anti-inflammatory outcome of disulfiram (DSF-Lf NPs) in DSS-treated mice as a model of ulcerative colitis. It is known that DSF acts as a potent inhibitor of the gasdermin-induced pore generation that causes pyroptosis and pro-inflammatory cytokine release. According to the findings, DSF-LF NP treatment prevented the body weight loss and colon length shortening, although this effect was also found with LF NPs. Importantly, DSF-Lf NPs did not exhibit toxic effects. Thus, DSF-LF NPs protected the damaged colonic epithelium and blunted the cell infiltration associated with DSS-induced colitis [66]. 

### 4.2. Colon Cancer

Colorectal cancer (CRC) is one of the principal causes of morbidity and mortality, and for its treatment, 5-Fluorouracil (5-FU) is prescribed as the standard chemotherapeutic agent [70]. Natural products such as bLf may provide beneficial properties against cancer as shown in in vitro studies of human colon carcinoma cell lines such as HT-29/B6 and T84 cells [71]. The results showed that preincubation with bLf in TNFα-primed colonic cell lines indicated that bLf attenuated the effect of TNFα on the loss of permeability and did not modify the increase in tight junction proteins. However, bLf decreased the cleavage of caspase-3, resulting in the blunting of apoptosis. These effects suggest bLf as a protective agent against the pro-inflammatory effects of TNFα on the barrier dysfunction in human colonic cancer. 

A high rate of tumor resistance and toxicity limits the efficacy of 5-FU and oxaliplatin [70]. For that reason, some research has been focused on the use of the Lf as a nanocarrier to drive the selectivity to epithelial intestinal cells through its receptor of some chemotherapeutic drugs [67]. In this regard, oxaliplatin (Lacto-Nano-Oxalo) and 5-FU (Lacto-Nano-5FU) were loaded in Lf nanoparticles prepared by a water-in-oil process and evaluated under in vitro and in vivo environments. In vitro culture of the human colon adenocarcinoma cell line COLO-205 evidenced that in comparison with free unloaded drugs, Lacto-Nano-Oxalo and Lacto-Nano-5FU NPs increased the uptake, remaining time, and antiproliferative activity and decreased the 50% inhibitory concentration (IC50) by less than 50%. In vivo evaluations in colon adenocarcinoma induced with azoxymethane in Wistar rats indicated that with regard to the free-drug-treated rats, intravenous Lacto-Nano-Oxalo and Lacto-Nano-5FU treatment prevented the body weight decrease, as a clinical manifestation of cancer, and decreased the number of colonic aberrant crypt foci and favored the colonic biodistribution. In order to assess the safety in Lacto-Nano-Oxalo- or Lacto-Nano-5FU-treated rats, toxicological analysis included liver toxicity by evaluating the serum levels of glutamic oxaloacetic transaminase (SGOT) and serum glutamic pyruvic transaminase (SGPT), renal toxicity assessed with creatinine and blood urea nitrogen (BUN), and hematopoietic toxicity (bone marrow suppression of subpopulation of white blood cells (WBCs) as neutrophils, lymphocytes, platelets, and red blood cells (RBCs)). In comparison with free drugs, both Lacto-Nano-Oxalo or Lacto-Nano-5FU did not show toxicity, and it was even like the control group treated with saline. These results allow the consideration of oxaliplatin and 5-Fluorouracil-loaded Lf nanoparticles as a safer delivery platform to manage the colon adenocarcinoma [67].

In other contributions, a delivery system was reported based on alginate-enclosed, chitosan-conjugated, iron-saturated bovine Lf (Fe-bLf) nanocarriers/nanocapsules (AEC-CCo-CP-Fe-bLf NCs) to assess its impact on cancer biomarkers in triple-positive CD133+, EpCAM+, and CD44+ Caco-2 colon cancer cell lines. These NCs were built with calcium phosphate nanocores loaded with Fe-bLf using electrostatic interaction, and then they were coated with 0.01% *w*/*w* of chitosan and 2% *w*/*w* of alginate [28]. The results in in vitro cultures of the Caco-2 colon cancer cell line indicated that in comparison with free Fe-bLf, AEC-CCo-CP-Fe-bLf NCs were efficiently internalized and caused an effective inhibition of tumor spheroid growth by decreasing the expression of survivin (an apoptosis inhibitor) and the expression malignancy markers on stem cells. In vivo assays were conducted in nude mice fed AEC-CCo-CP-Fe-bLf supplemented with an AIN 93G pellet diet after their inoculation with Caco-2 cells to develop the tumor after 35 days. The findings evidenced that AEC-CCo-CP-Fe-bLf NC treatment enabled an effective internalization of Fe-bLF through Divalent Metal Transporter 1 (DMT1), lipoprotein receptor protein (LRP), transferrin receptor (TFR), and Lf receptor (LfR) along with the antitumor efficacy reducing the viability of Caco-2 cells. Furthermore, in athymic nude mice fed AEC-CCo-CP-Fe-bLf NCs in a pellet diet showed a down-regulated mRNA expression of antiapoptotic factors including survivin and the Bcl-2 family, whereas mRNA up-regulated the expression of proapoptotic markers such as Bax, Fas, and caspase-9 and -3. In addition, AEC-CCo-CP-Fe-bLf NCs in the diet reduced the cancer markers of malignity of the Caco-2 cell (CD3133, EpCAM, and CD44). A critical outcome of this delivery system was the lack of nanotoxicity, which thus provided both effectivity and safety. Presumable mechanisms of AEC-CCo-CP-Fe-bLf NCs seem to encompass different types of miRNA related to iron regulation in different organs (miRNA-lET7D: the brain; miR485: the intestine; miR210: the liver; and miR196: the intestine). The data support the outcome of AEC-CCo-CP-Fe-bLf NCs in maintaining the iron-free levels to prevent oxidative stress [28]. 

In other models, nanocapsules were formulated with zinc ferrite (Zn-Fe) and iron-saturated bLf (Zn-Fe-bLf) complexes coated with chitosan and alginate nanogel [68]. In vitro cultures of triple-negative breast cancer cells (MDA-MB-231) indicated that with regard to the void NCs, Zn-Fe-bLf NCs were efficiently internalized in 2 h and showed a dose response regarding colony growth inhibition. In vivo studies were performed in athymic BALB/c nu/nu mice fed Zn-Fe-bLf NCs supplemented with AIN 93G, and thereafter, the tumor was established by administering Caco-2 human colon carcinoma or breast (MDA-MB-231) cancer cells. The findings evidenced that in comparison with vehicle, Zn-Fe-bLf NCs decreased the tumor volume and caused a complete regression in tumor volume after 90 days of feeding in both tumor models. Furthermore, Zn-Fe-bLf NCs showed tumor-site-specific localization evaluated by near-infrared fluorescence imaging in living mice; the last effect also was observed ex vivo. Finally, Zn-Fe-bLf NCs can be considered as safe, with eco-friendly characteristics, nontoxicity, and high biocompatibility [68].

The anticancer activity of the fully Fe^3+^-saturated bLf (AEC–CP–Fe-bLf) and paclitaxel (Taxol^®^ Bristol Meyers Squibb New York USA) (AEC–CP–Taxol) adsorbed onto calcium phosphate nanocores, enclosed in biodegradable polymers chitosan and alginate, was tested in in vitro and in vivo assays [69]. In vitro culture assays of the Caco-2 cell line indicated that both AEC–CP–Fe-bLf and AEC–CP–Taxol NC enhanced endocytosis and transcytosis and increased apoptosis and cytotoxicity. In vivo assays were conducted in BALB/c mice fed AEC-CP-Fe-bLf supplemented with an AIN 93G pellet diet before or after their inoculation with Caco-2 cells to develop the tumor. The data indicated that with regard to mice fed the normal AIN 93G diet, mice fed AEC-CP-Fe-bLf supplemented with the AIN 93G diet in both preventive and therapeutic modalities did not develop tumor growth, nor did they show toxicity signs. Furthermore, AEC–CP–Fe-bLf induced the regression of tumors and had low toxicity on other no-target tissues. However, AEC–CP-Fe-bLf NCs enabled the release of Fe-bLf, protected it from the harsh conditions of the gut environment, and enhanced the bioavailability and metabolic stability of Fe-bLf [69].

**Figure 2:**
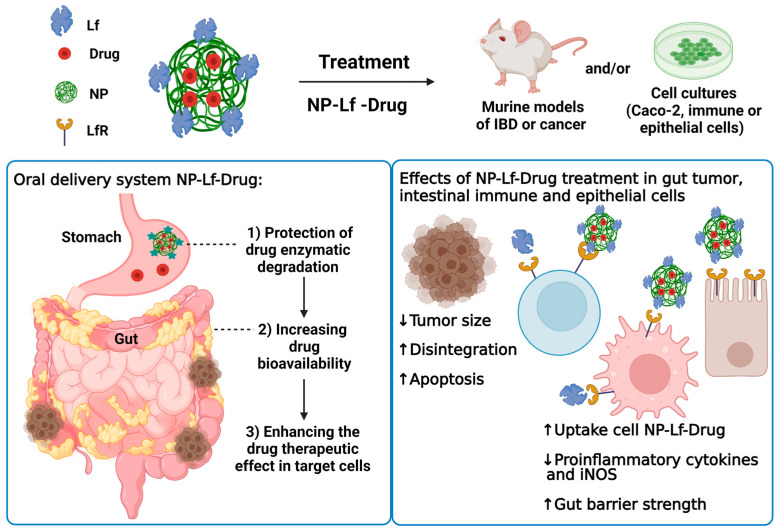
Lactoferrin products and intestinal therapeutic effects in tumor, immune, and epithelial cells. Created in Biorender.com. accessed on 24 January 2023.

Accordingly, this evidence supports the beneficial activity of Lf in pharmaceutical products with prophylactic and therapeutic potential. These products may be a safe approach for enhancing the pharmacological effect and decreasing the tolerance of conventional drugs used for IBS and colon cancer. Figure 2 depicts Lf preparations with potential therapeutic action for IBD. 

## 5. Neurodegenerative Diseases

The blood–brain barrier (BBB) is a polarized monolayer composed of microvascular endothelial cells sealed by tight junctions that encompasses an apical membrane faced to the bloodstream and basolateral membrane faced to brain tissue [72]. The BBB has specific characteristics that restrict the passage of substances to the central nervous system (CNS), which is a limitation for pharmacological treatment in some neurodegenerative diseases, including Alzheimer’s, Parkinson’s, multiple sclerosis, and epilepsy, among others [73]. For this reason, work is currently being carried out on the design of effective delivery systems using molecules whose physicochemical properties allow drugs to be transported within the CNS. Some therapeutic compounds have been arranged in delivery systems based on nanoparticles composed of biodegradable polymers such as polyethylene glycol (PGE), lactic acid (PLA), and chitosan (CS). These components have been shown to decrease toxicity, to control release, to improve drug bioavailability and therefore their therapeutic efficacy [74]. However, these delivery systems have some limitations; for example, the charge and the size of the particles can prevent interaction with the cells of the BBB. For this reason, molecules that function as carriers that can cross the BBB through a process known as transcytosis are added to these release systems. Transcytosis refers to the transport of macromolecules from the apical to the basolateral membrane of endothelial cells, and the process may be mediated by receptors. The BBB expresses receptors for lipoproteins or insulin that can carry out transcytosis for their corresponding ligands and even modulatory proteins such as Lf [72].

The low-density lipoprotein receptor-related protein 1(LRP1) is one of the Lf receptors and is widely expressed in the BBB [75]. The union of Lf with the receptor allows the phenomenon of transcytosis [72]. Thus, in recent years the design of release systems with Lf added has been a great technological advance so that molecules with therapeutic activity such as drugs, proteins, or genetic material can reach the CNS. These preparations have been tested in some in vitro and/or in vivo models of Alzheimer’s and Parkinson´s disease as summarized in Table 3.

### 5.1. Alzheimer’s Disease

Alzheimer’s disease (AD) is one of the CNS diseases characterized by memory loss, and the cholinergic system is, by far, the most important in the development of the disease. Neurodegenerative and inflammatory conditions are present in this disease. The pathological features of AD include β-amyloid extracellular plaques, intracellular hyperphosphorylated aggregates of tau protein (NFTs), oxidative stress, and an abnormal apoptotic cascade in the susceptible brain regions (the cerebral cortex and the hippocampus) [84]. Apoptosis requires various crucial factors that include apoptotic and antiapoptotic factors B cell lymphoma 2 (Bcl2), caspases, TNFα, Bcl2-associated X protein (Bax), and reactive oxygen species production; all of these influence AD progression [84]. To improve the therapeutic effect against AD, some novel products such as nanoemulsions (NE) have been formulated with Lf as a carrier of herbal components of the Chinese traditional medicine with neuroprotective action, such as Huperazine (alkaloid) α-asarone, bacosides (terpenoids), and Thanshinone (flavonoid). Experimental assays evidenced that these emulsions improved the availability of herbal compounds in some areas of the CNS, and in vitro assays documented that NE enhanced the cell uptake in endothelial cell cultures; in addition, NE did not cause toxicity [75,76,77].

Huperazine is an acetylcholinesterase inhibitor and in animal models has been shown to improve memory through different neuroprotective mechanisms. However, the physicochemical properties of this drug make it poorly available to the CNS. In vitro studies have been conducted to analyze the Huperazine loaded in Lf nanoemulsions (NEs) based on soybean oil [78] and polylactic-co-glycoside (PLGA) [33]. Findings from cultures of the human cerebral microvascular endothelial cell (hCMED/D3) cell line and human neuroblastoma SH-SY5Y cell line showed that the uptake of Lf nanoemulsions loaded with huperazine was more efficient than the cell uptake of the pure drug [33,78]. Experimental results of in vivo fluorescence studies in rodents indicated that after the intranasal administration of Lf-NEs loaded with huperazine, the accumulation of these Lf-Hup-NEs increased in some areas of the CNS compared with the administration of the free drug or in emulsion without Lf [33,78].

Scopolamine is a non-selective competitive antagonist of muscarinic cholinergic receptors used to induce cognitive deficits associated with dementia and aging. Bacosides are terpenic compounds that act as acetylcholine cholinesterase inhibitors and therefore increase the levels of acetylcholine and are used as neuroprotectors. In an animal model of Alzheimer’s scopolamine induced in Swiss albino mice, it was found that the intravenous administration of nanoparticles in polymerosomes composed of polyethylene glycol-polylactic acid-polycaprolactone (PEG-PLA-PLC) loaded with bacosides and conjugated with Lf reversed the memory loss induced by scopolamine, but this effect did not occur when the bacoside extract was administered without the delivery system [77]. 

Several studies have demonstrated the neurotoxic potential of aluminum in different experimental animal models. Aluminum is a risk factor in the etiology and pathology of different neurodegenerative disorders such as Alzheimer’s disease and dementia associated with Parkinson’s disease since it induces conformational changes in key proteins in the pathophysiology of these diseases [85]. An experiment regarding Alzheimer’s disease in Swiss mice induced with aluminum chloride (AlCl_3_) tested polyamidoamine-type dendrimers (PAMAM) conjugated with Lf (Lf-PAMAM) nanoparticles and loaded with memantine (MEM), a noncompetitive antagonist of N-methyl-D-aspartate (NMDA), a glutamate receptor, and used for AD therapy. The intraperitoneal administration of Lf-MEM-PAMAN attenuated the memory loss compared with the administration of memantine in nanoparticles without Lf [79]. 

It is known that free iron participates in the generation of reactive oxygen species causing neuronal damage and apoptosis. Likewise, the neuroprotective effects of deferasirox (a high-affinity chelator iron) in carbodiimide nanoconjugates with bLf were evaluated in an animal model of Alzheimer’s in Wistar rats induced by the administration of peptide β-amyloid at the hippocampal site. The intraperitoneal administration of bLf-nanoconjugates (NC) loaded with deferasirox attenuated the learning deficit through mechanisms that involved the decrease in the expression of proteins such as caspase 3, Bax, and Bcl2. In vitro assays in the rat pheochromocytoma (PC12) cell line primed with hydrogen peroxide as an apoptotic inducer evidenced that the Lf–deferasirox conjugate prevented the toxicity caused by hydrogen peroxide in endothelial cell cultures [80]. 

Another delivery system was based on polyethylene glycol-co-glycolic acid (PEG-PLGA) polymerosomes (POS) containing Lf and loaded with ST4G-humanin (SHN) protein. The latter is a neuroprotective peptide very prone to proteolytic enzymatic degradation. An animal model of Alzheimer’s in rats induced by administration of peptide β-amyloid at a hippocampal site showed that the intravenous administration of POS-Lf-SHN favored the entry of SHN to the brain compared with the administration of the POS-SHN carrier system without Lf, as shown by the area under the curve (AUC) assay [73]. Furthermore, treatment with POS-SHN-Lf attenuated the expression of Bax and caspase 3 proteins. The data suggested neuroprotective effects of the POS-Lf–SHN delivery system to prevent apoptosis and improve therapeutic effect of SHN in Alzheimer’s disease.

The design of nanocarrier systems loaded with microRNA (miRNA) has been very useful in the development of new treatments against Alzheimer’s. It has been shown that the β-amyloid peptides are derived from amyloid precursor protein (APP) by the site APP-cleaving enzyme-1 (BACE-1) and secretase; therefore, the BACE-1 inhibition is a therapeutic strategy for disease control. In a recent study, a delivery system based on stearic acid (SA), polyethyleneimine (PEI), or chitosan (CS) and loaded with Lf was designed to carry a recombinant pre-miR-29b (microRNA) to block the BACE-1 protein expression. The results from in vitro experiments indicated that both carrier systems miRNA-CS-SA-Lf and miRNA-PEI-SA-Lf increased their intracellular reuptake in the rat brain microvascular endothelial (RBE4) cell line and silenced the expression of the human BACE gene better than the negative control that only contained the miRNA without Lf [81]. 

### 5.2. Parkinson’s Disease

Parkinson’s disease is a neurodegenerative disorder characterized by the deterioration of dopaminergic neurons at the nigrostratial region, which causes decreased level of dopamine in the striatum and leads to abnormal motor control [86]. Various animal models using toxins have been used to study the pathophysiology of Parkinson’s. Intracerebral administration of 6-hidroxy-dopamine (6-OHDA) directed to the striatum rapidly oxidizes and produces reactive oxygen species such as hydrogen peroxide, superoxide radicals, and hydroxyl radicals that lead to mitochondrial dysfunction. For instance, 6-OHDA injection will damage the axon terminals first in the striatum, followed by dopaminergic neuron degeneration in the substantia nigra (SN) [86].

Experimental studies of Parkinson´s disease showed that nanoparticles modified with bLf loaded with dopamine (Lf-DOP-NP) [82]) or rotigotine, a non-ergolinic dopamine agonist (Lf-ROT-NP) [83], increased the cell uptake evaluated in SH-SY5Y and 16HBE cell cultures compared with nanoparticles without bLf [74]. In vivo studies show that the intranasal administration of the Lf-DOP-NP or Lf-ROT-NP formulations decreased the contralateral rotations, as seen in the model of Parkinson’s in rats intracerebrally injected with 6OHDA. The presumable mechanism involved the increase in the expression of dopamine transporter (DAT) and of tyrosine hydroxylase enzyme protein [82,83].

**Figure 3:**
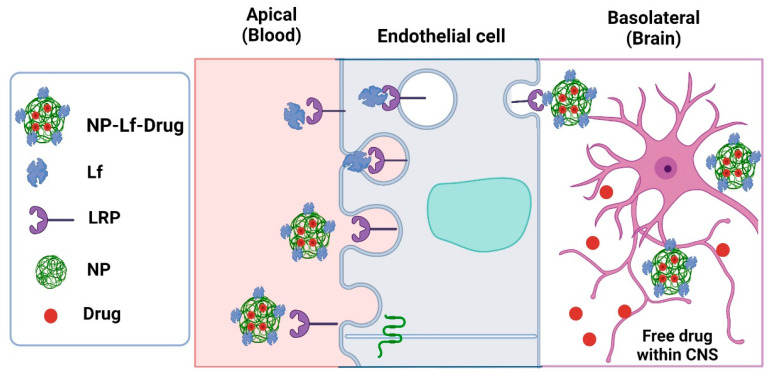
Lactoferrin formulations as a carrier of drugs to cross the endothelial cells of the BBB through the phenomenon of transcytosis using its receptor low-density lipoprotein receptor-related protein (LRP) in nanoparticles drug delivery systems (NP-Lf-Drug). Created in Biorender.com. accessed on 24 January 2023.

These studies show that Lf used in novel delivery systems improves the availability of molecules to the CNS. Moreover, the study of these delivery systems provides evidence to support signaling mechanisms of drugs for the treatment of neurodegenerative diseases such as Alzheimer’s and Parkinson’s. The overview of the Lf delivery systems of drugs for neurodegenerative disease is depicted in Figure 3.

## 6. Conclusions

The antimicrobial properties of Lf have propelled its use in pharmaceutical products. Formulations containing Lf as a natural antimicrobial agent or as an antimicrobial carrier may be a promising and sustainable strategy. As mentioned previously, most knowledge has arisen from experiments with promising results; however, future studies in human trials are needed to confirm the effectivity and the toxicity of the Lf products. 

Additionally, the gastrointestinal tract expresses various Lf receptors; therefore, Lf-added delivery systems have been designed to transport and protect drugs from enzymatic degradation in the intestine favoring the bioavailability to target organs for the treatment of inflammatory bowel disease and colon cancer. 

As described in this review, Lf has also been used to design nanoparticle delivery systems that transport drugs, which cannot cross the blood–brain barrier, within the central nervous system. In this way, many molecules with therapeutic purposes are being tested in these formulations, and in the future, they may contribute to the treatment of neurological diseases such as Alzheimer’s and Parkinson’s. 

The findings provide insights about the therapeutic possibilities of pharmaceutical Lf preparations with a sustainable approach that contributes to decreasing the resistance of antimicrobials and improving the bioavailability and therapeutic effects of first-line drugs for chronic diseases. Information concerning substantive mechanisms, bioavailability, and/or toxicity is not fully known, but these issues may represent future challenges for their therapeutic optimization for human infections. 

## Figures and Tables

**Table 1:** Pharmaceutical antimicrobial bLf formulations.

Product	Test	Ref
Antibacterial		
Alginate gel-encapsulated ceramic nanocarriers loaded with 1.2% iron-saturated bLf (EAC-CP-Fe-bLf NCs)	BALB/c orally infected with *Salmonella typhimurium* and then fed EAC-CP-Fe-bLf NCs in diet	[38]
Hydroxyapatite nanoparticles coated with lactoferrin (Lf-HA NPs)	BALB/c orally infected with *Helicobacter pylori* and then orally treated with Lf-HA NPs alone or plus cell free supertant of *Lactobacillus paracasei* culture	[39]
Multilamellar liposomes prepared with soy phosphatidylcholine containing 180 mg of bLf	Human volunteers with periodontal infection treated with bLf-liposomes (4 tablets per day) during 0, 2, and 4 weeks	[40]
Antiprotozoan		
Alginate gel-encapsulated ceramic nanocarriers loaded with 1.2% iron-saturated bLf (EAC-CP-Fe-bLf NCs)	BALB/c infected via intraperitoneal with *Toxoplasma gondii* tachyzoites and then fed EAC-CP-Fe-bLf NCs in diet	[27]
Nanoparticles formulated with bLf adsorbed over core poly-D,L, lactide -co-glicolide loaded with amphotericin B (LcfPGNP-Amb)	Hamsters intraperitoneally (ip) infected with *Leishmania donovani* amastigotes and then treated intraperitoneally with 1 mg/kg via ip for 5 consecutive days with Amb encapsulated in LcfPGNP	[41]
Betulinic acid loaded within poly-D,L,co-glicolide acid nanoparticles coated with bLf (Lf-BANPs)	BALB/c-derived macrophages infected with *Leishmania donovani* amastigotes treated with Lf-BANPs (2.5 μg/mL)	[42]
Alginate-enclosed chitosan-conjugated calcium phosphate nanocapsules with 1.2% *w*/*w* iron-saturated buffalo Lf (AEC-CCo-CP-buLf-NCs)	BALB/c mice infected intraperitoneally with parasitized erythrocytes from Swiss mice infected via ip with *Plasmodium berghei* and fed AEC-CCo-CP-buLf-NCs in diet	[43]
Antiviral		
Human recombinant iron-saturated Lf loaded in silver and gold nanoparticles (Lf-Ag-Au NPs)	Female C57BL/6 mice intravaginally infected with herpes simplex virus plaque forming units (PFU) and thereafter washed with 10 μg/mL of Lf-Ag-Au NPs in saline solution	[44]
Zidovudine (AZT) loaded in Lf nanoparticles prepared with olive oil (AZT-Lacto-Nano)	Pharmacokinetic and toxicologic assessment in blood, liver, and kidney of Wistar rats orally administered with AZT-lacto-Nano	[45]
Efavirenz (anti-HIV) curcumin (antimicrobial spermicide) loaded in Lf nanoparticles (ECLNPs)	Pharmacokinetic, toxicologic, and vaginal Lactobacillus assessment in vaginal lavage of female Wistar rats intravaginally administered with ECLNPs	[46]
Efavirenz, curcumin, and Lf nanoparticles (ECLNPs)	Reproductive performance, fertility, and postnatal development in female Wistar rats intravaginally or orally treated with ECLNPs	[47]
Tenofovir (anti-HIV) and curcumin loaded in Lf nanoparticles (TCNPs)	Pharmacokinetic and toxicologic assessment of ECLNPs in vaginal epithelium of rats	[48]

**Table 2:** Products for intestinal dysfunctions.

Product	Test	Ref
Intestinal Bowel Disease		
Emodin entrapped in Lf nanoparticles and then loaded in yeast wall microparticles (EMON-YPs)	DSS-induced colitis in BALB/c mice orally treated with EMON-YPs	[22]
Lf NPs formulated with calcium pectinate and hyaluronic acid as oral carriers of rhein (RH) as anti-inflammatory herbal compound (CP-HA-RH-NPs)	DSS-induced UC in mice orally treated with CP-HA-RH-NPs	[23]
Disulfiram-loaded Lf nanoparticles (DSF-Lf-NPs)	DSS-induced colitis in BALB/c mice intravenously injected with DSF-Lf NPs	[66]
Colorectal Cancer		
Alginate-enclosed chitosan-conjugated iron-saturated bLf nanocapsules (AEC-CCo-CP-Fe-bLf-NCs)	Athymic nude mice fed AEC-CCo-CP-Fe-bLF-NCs in diet	[28]
Lactoferrin nanoparticles loaded with Oxaliplatin (Lacto-Nano-Oxalo) and 5-fluorouracil (Lacto-Nano-5FU)	Adenocarcinoma induced with azoxy-methane in Wistar rats intravenously injected with Lacto-Nano-Oxalo or Lacto-Nano-5FU (40 mg/Kg body weight, four doses in four consecutive weeks)	[67]
Zinc-ferrite (Zn-Fe) and iron-saturated bLf (Zn-Fe-bLf) complexes coated with chitosan and alginate nanogel (Zn-Fe-bLf-NCs)	Human xenograft colonic adenocarcinoma model in athymic nu/nu BALB/c mice fed Zn-Fe-bLf-NCs in AIN 93G diet	[68]
Alginate-enclosed chitosan calcium phosphate loaded on iron-saturated bLf nanocarriers (AEC-CP-Fe-bLf NCs)	Human xenograft colonic adenocarcinoma model in athymic nu/nu BALB/c mice fed Zn-Fe-bLf-NCs in AIN 93G diet	[69]

**Table 3:** Lactoferrin products for neurodegenerative diseases.

Product	Test	Ref
Alzheimer’s		
α-asarone-loaded Lf-NPs	Sprague–Dawley rats intranasally treated with Lf-NPs to assess pharmacokinetics proprieties, distribution in the brain and other tissue, brain targeting, and toxicity	[75]
Tanshinone I (TSI) nanoemulsion (TSI-NE) modified with Lf	In vitro assays for evaluating the uptake of TSI-Lf-NE by mouse brain microvascular endothelial cell line (bEnd.3 cells) using Coumarin-6 as a fluorescent probe	[76]
Bacoside-loaded-Lf-conjugated polymersomes	In vivo assays of Alzheimer’s model induced by scopolamine in male Swiss albino mice treated via tail vein with bacoside-loaded-Lf-conjugated polymersomes for evaluating uptake of the polymersomes in the brain assayed by LCMS assay	[77]
Huperzine A (HupA)-loaded NPs with surface modification by Lf-conjugated nanoemulsions (NEs)	Rats intranasally administred with HupA-NEs, Lf-HupA-NEs, and HupA solution to investigate the brain-targeting effects of these formulations	[78]
Huperzine A (HupA)-loaded NPs with surface modification by Lf conjugate	In vivo intranasally administered Hup-A NPs after intranasal administration in KM mice for evaluating the biodistribution	[33]
Dendrimers (PAMAM) and Lf conjugate loaded with memantine (MEM).	The in vivo study in AlCl_3_-induced Alzheimer’s (AD) in Swiss albino mice treated with MEM-PAMAM-Lf intraperitoneally; in vivo biodistribution in the Sprague−Dawley rat model for the brain uptake of MEM-PAMAM-Lf (formulations administrated intraperitoneally)	[79]
Deferasirox–Lf conjugated	Rat model of Alzheimer’s disease induced by beta amyloid and treated with the deferasirox–Lf conjugated intraperitoneally for evaluating cognitive disorder	[80]
Poly(ethyleneglycol)-poly (D,L-lactic-co-glycolic acid) (PEG-PLGA) polymersomes conjugated with Lf (Lf-POS); S14G-humanin conjugate- Lf-POS	In vivo model of Alzheimer’s disease in Sprague–Dawley rats induced with hippocampal administration of Amyloid-β25–35 and treated with Lf-POS (SHN-Lf-POS) for evaluating the neuroprotective effects	[73]
Polymers of chitosan and polyethyleneimine were loaded with recombinant precursor microRNA (pre-miR-29b) and Lf	In vitro assays in N2a695 and RBE4 cell lines incubated with pre-miR-29b-loaded polyplexes to assess expression of BACE-1	[81]
Parkinson’s		
Lactoferrin co-modified nanoparticles (Lf-BNPs) encapsulated dopamine	In vivo model of Parkinson´s disease induced by 6-hydroxydopamine in Sprague–Dawley rats, intranasally administrated with dopamine Lf-BNPs for evaluation of the effects on behavior; in vitro uptake and cytotoxicity studies in SH-SY5Y and 16HBE cells	[82]
Lactoferrin-modified rotigotine nanoparticles (Lf-R-NPs)	Intranasal administration of Lf-R-NPs in mice for assay biodistribution; qualitative and quantitative cellular uptake for evaluating the accumulation of Lf-NPs in 16HBE and SH-SY5Y cells	[74]
Lactoferrin-modified rotigotine nanoparticles (Lf-R-NPs)	In vivo model of Parkinson’s disease induced by by nigrostriatal administration of 6-hydroxydopamine in Sprague−Dawley rats intranasally treated with Lf-R-NPs for evaluating contralateral rotations and biodistribution	[83]

## Data Availability

Data sharing not applicable.
